# Two-Level Corpectomy and Fusion vs. Three-Level Anterior Cervical Discectomy and Fusion without Plating: Long-Term Clinical and Radiological Outcomes in a Multicentric Retrospective Analysis

**DOI:** 10.3390/life13071564

**Published:** 2023-07-14

**Authors:** Giorgio Lofrese, Sokol Trungu, Alba Scerrati, Pasquale De Bonis, Francesco Cultrera, Lorenzo Mongardi, Nicola Montemurro, Amedeo Piazza, Massimo Miscusi, Luigino Tosatto, Antonino Raco, Luca Ricciardi

**Affiliations:** 1 Neurosurgery Unit, Bufalini Hospital, 47521 Cesena, Italy; giorgio.lofrese@gmail.com (G.L.); francesco.cultrera@auslromagna.it (F.C.); luigino.tosatto@auslromagna.it (L.T.); 2 NESMOS Department, “Sapienza” University of Rome, Sant’Andrea Hospital, 00185 Rome, Italy; sokol.trungu@uniroma1.it (S.T.); amedeo.piazza@uniroma1.it (A.P.); massimo.miscusi@uniroma1.it (M.M.); antonino.raco@uniroma1.it (A.R.); 3 Neurosurgery Unit, Cardinale G. Panico Hospital, 73039 Tricase, Italy; 4 Department of Neurosurgery, S. Anna University Hospital, 44124 Ferrara, Italy; scrlba@unife.it (A.S.); dbnpql@unife.it (P.D.B.); lorenzo.mongardi@auslromagna.it (L.M.); 5 Department of Neurosurgery, University of Pisa, 56126 Pisa, Italy; nicola.montemurro@unipi.it

**Keywords:** anterior cervical discectomy and fusion, anterior cervical corpectomy and fusion, cervical spondylotic myelopathy, discectomy, corpectomy, fusion, ACDF, ACCF

## Abstract

Background: Anterior cervical discectomy and fusion (ACDF) and anterior cervical corpectomy and fusion (ACCF) represent effective alternatives in the management of multilevel cervical spondylotic myelopathy (CSM). A consensus on which of these techniques should be used is still missing. Methods: The databases of three centers were reviewed (January 2011–December 2018) for patients with three-level CSM, who underwent three-level ACDF without plating or two-level ACCF with expandable cage (VBRC) or mesh (VBRM). Demographic data, surgical strategy, complications, and implant failure were analyzed. The Neck Disability Index (NDI), the Visual Analog Scale (VAS), and the cervical lordosis were compared between the two techniques at 3 and 12 months. Logistic regression analyses investigated independent factors influencing clinical and radiological outcomes. Results: Twenty-one and twenty-two patients were included in the ACDF and ACCF groups, respectively. The median follow-up was 18 months. ACDFs were associated with better clinical outcomes at 12 months (NDI: 8.3% vs. 19.3%, *p* < 0.001; VAS: 1.3 vs. 2.6, *p* = 0.004), but with an increased risk of loss of lordosis correction ≥ 1° (OR = 4.5; *p* = 0.05). A higher complication rate in the ACDF group (33.3% vs. 9.1%; *p* = 0.05) was recorded, but it negatively influenced only short-term clinical outcomes. ACCFs with VBRC were associated with a higher risk of major complications but ensured better 12-month lordosis correction (*p* = 0.002). No significant differences in intraoperative blood loss were noted. Conclusions: Three-level ACDF without plating was associated with better clinical outcomes than two-level ACCF despite worse losses in lordosis correction, which is ideal for fragile patients without retrovertebral compressions. In multilevel CSM, the relationship between the degree of lordosis correction and clinical outcome advantages still needs to be investigated.

## 1. Introduction

Cervical spondylosis is a common cause of neurologic morbidity, consisting of myelopathy and radiculopathies. Whether ventral compression or segmental kyphosis represents the radiological scenario, the anterior cervical decompression and fusion procedure is often recommended [1,2,3]. In the case of three-level contiguous spondylosis, the treatment strategy for decompressing the spinal cord and reconstructing the anterior column is still debated. On one hand, three-level anterior cervical discectomy and fusion (ACDF) shows an increased risk of incomplete decompression and nonunion because of multiple graft–host interfaces [4,5,6,7,8], but with a low rate of implant failures. On the other hand, with two-level anterior cervical corpectomy and fusion (ACCF), a higher risk of instrument-related complications, spinal cord/nerve root injuries, and excessive bleeding is reported [7,9,10,11,12,13], but the fusion rate improves, and more extensive decompression is provided. Many studies in the literature address the comparison between discectomy and cervical corpectomy in CSM [7,8,9,10,11,12,13,14], but only a few of them compare two-level ACCF with three-level ACDF [15,16,17]. Therefore, there is no consensus on how to properly select patients who may benefit the most from each technique [18,19,20]. This study aimed to evaluate and compare the long-term clinical and radiological outcomes of two-level ACCF and three-level ACDF without plating, distinguishing double corpectomies with mesh from those with expandable cages.

## 2. Materials and Methods

### 2.1. Study Design

Three Italian centers contributed to the present retrospective study, in accordance with the WMA Declaration of Helsinki: Sant’Andrea University Hospital of Rome; Sant’Anna University Hospital of Ferrara; Bufalini Hospital–Cesena. The institutional databases of the contributing centers were screened for eligible patients to be included in the study with a search set from January 2011 to December 2018. Informed consent was obtained from all participants, and data were retrieved and extracted anonymously. The Ethics Committee approval number is reported on the title page for double-blind review.

### 2.2. Study Population and Inclusion Criteria

Inclusion criteria were as follows: patients suffering from cervical myelopathy primarily determining their clinical signs and symptoms; patients with spinal cord compression or signal changes due to multilevel disc herniations and/or osteophytes; patients with cord compression or signal changes due to multilevel hypertrophied PLL or the segmental type of ossification of the posterior longitudinal ligament (OPLL); and patients undergoing three-level ACDF without plating or two-level corpectomy. Patients with a history of spinal trauma, with previous cervical spine surgery, or with missing/incomplete data (clinical/radiological) were excluded from the study. Positive cancer history, osteoporosis treatment, and former osteoporotic vertebral fractures were other exclusion criteria, together with radiological evidence of a circumferential cervical spinal stenosis or a concomitant posterior fusion.

### 2.3. Surgical Technique

Before surgery, clinical evaluation along with MRI, CT scan, and radiographic studies of the cervical spine were performed. All the patients were operated on using the traditional Smith–Robinson approach to the subaxial cervical spine [21]. Blunt dissection was performed with Cloward retractors, medial to the sternocleido-mastoid muscle (SCM) and neurovascular bundle (NVB) of the neck: internal carotid artery (ICA), internal jugular vein, and vagal nerve (X CN) with its overlying pretracheal fascia. The approach runs lateral to the trachea and esophagus; the prevertebral plane was then accessed after opening the pretracheal fascia medial to the NVB and being careful not to injure the midline structures (trachea and esophagus). The prevertebral fascia was incised longitudinally; the longus colli muscle was identified and dissected subperiosteally and laterally once the prevertebral space was accessed. The choice of operation was dependent on the characteristics of cord compression. The segments with retrovertebral compressive pathology, such as large endplate osteophytes and huge prolapsed intervertebral discs, were decompressed via corpectomy, while those with soft disc herniations were decompressed via discectomy. Operations were performed by the same experienced spine surgeon at each individual institution.

### 2.4. Surgical Data

The following surgical data were recorded: type of skin incision (horizontal/oblique); surgical strategy (two-level ACCF with plating/three-level ACDF without plating); and implant used (interbody cage–ACDF/expandable vertebral body replacement cage–VBRC/vertebral body replacement mesh–VBRM). Both titanium expandable cages and meshes were always filled with autologous bone from the corpectomy, and different from the three-level ACDF with tantalum cages, an anterior plate always completed the two-level ACCF instrumentation. The tantalum cages in the ACDF group and the anterior plates in the ACCF group were the same brand and model in all the respective cases. Surgical duration (min), intraoperative blood loss (mL), intraoperative evidence of a violation of the endplate, and complications were also recorded. Because of the retrospective design of the study, with several surgeons involved from different centers, there was no standardized protocol addressing surgery on a three-level ACDF rather than a two-level ACCF. Nevertheless, this latter strategy was always used when facing myelopathy sustained by a significant retrovertebral compression, such as an OPLL, or by deforming cervical spondylosis. In all patients undergoing ACCF, a soft collar for pain relief was prescribed for two weeks. 

### 2.5. Clinical Outcome

At admission, each patient was clinically assessed with the ASA score, the Neck Disability Index (NDI-pre), and the Visual Analog Scale (VAS-pre) to evaluate, respectively, general physical status, neck-related disability, and chronic pain scores. Diabetes and smoking status were registered as factors influencing bone mineral density and implant-related complications. Days of hospital stay, perioperative complications, and reinterventions were also analyzed. All patients were followed up as outpatients at 3 (-post) and 12 (-fup) months. The clinical outcome was assessed with the Neck Disability Index and the Visual Analog Scale, both administered on outpatients at 3 months (-post) and 12 months (-fup). The differences between -pre, -post, and -fup values were calculated using the “post-pre” and “fup-post” indexes, respectively, to assess the postoperative outcome at 3 months compared with the preoperative evaluation and the outcome evolution between 3 and 12 months, both for NDI and VAS.

### 2.6. Radiological Outcome

Preoperative MRI and cervical spine X-rays were independently evaluated by two expert spine surgeons (G.L. and L.R.), registering the presence of myelomalacia and measuring C2–C7 lordosis (CL) for each patient. The cervical lordosis was assessed using the Cobb angles of C2–C7 and fused segments. The former was formed by lines along the inferior endplate of C2 to the inferior endplate of C7 in a neutral position, and the latter was formed by lines along the superior endplate of the cephalad vertebral body and along the inferior endplate of the caudal vertebral body of the fused segments. The radiological outcome was evaluated using 3-month and 12-month cervical spine X-rays, calculating CL modification over time: the difference between CL-post and CL-pre was considered the lordosis correction, while the difference between CL-fup and CL-post helped in estimating the eventual loss of lordosis correction at the follow-up. Each value analyzed was the mean of the two values measured by the two operators. Implant subsidence and positioning at risk of mechanical failure were registered, while no cases of residual mobility were documented. 

### 2.7. Statistical Analysis

The statistical analyses were performed with MedCalc, version 15.4 (1993–2015 MedCalc Software bvba). Testing of the significance of changes in demographic data, ASA, smoking status, diabetes, myelopathy, type of incision, surgical strategy, implant used, complications in relation to surgical duration, intraoperative blood loss, and hospitalization, together with functional and radiological outcomes, was performed with repeated measurements of analysis of variance (ANOVA) and Chi-squared tests for categorical variables. Scheffé post hoc tests were performed for all the ANOVA tests. Age, ASA, hospitalization, and pre- and postoperative functional and radiological parameters were dichotomized whenever appropriate to determine the role of the different variables in positively or negatively influencing outcomes (clinical/radiological). Analysis of contingency tables was performed to investigate the relationships between patients’ demographic, diabetes, smoking status, myelopathy, type of incision, type of surgery, intra-/postoperative complications, and reinterventions with clinical and radiological outcomes. Logistic regression analysis examined the impact of the aforementioned parameters on the following dichotomized variables: VAS-post (≤4 or >4), VAS-fup (≤2 or >2), and loss of lordosis correction (0° or ≥1°). Results presenting *p* ≤ 0.05 were considered statistically significant.

## 3. Results

### 3.1. Study Population

Among the 49 patients eligible for this study, 2 died and 4 were lost at follow-up. In most of the remaining 43 patients, ASA 2 (62.8%) and myelopathy (86%) were documented, and only a minority reported smoking (37.2%) or diabetes (25.6%). The mean preoperative NDI and VAS were, respectively, 54.8% and 7%, without differences stratifying for gender and age. Similarly, the mean preoperative cervical lordosis was 7.67°. Hospitalization was significantly shorter after three-level ACDF than after VBRM/VBRC (3 days instead of 11 days), with mean hospital stays shorter for VBRM (5 days) than for VBRC (15 days) (F [2, 40] = 4.401, *p* = 0.02), because of two patients in this latter group exceeding 50 days. The median follow-up was 18 months (min–max: 12–91 months) (Table 1 and Table 2). 

### 3.2. Surgery

A double corpectomy was performed in 51.2% of the cases, with a VBRC used on 13 patients (30.2%). Three-level ACDFs were used in the remaining 48.8% of patients. An oblique incision was adopted in 90.7% of cases, contributing to shortened surgical times compared with a horizontal one (146 min instead of 310 min) (F [1, 41] = 16.327, *p* < 0.001). A double corpectomy was used on those between 50 and 59 years and three-level ACDF was used on those between 60 and 69 years. The mean surgical durations were 104, 186, and 235 min, respectively, for three-level ACDFs, two-level ACCFs with VBRM, and two-level ACCFs with VBRC (F [2, 40] = 14.595, *p* < 0.001). No significant differences were noted in terms of intraoperative blood loss, with mean values of 100 cc and 91 cc, respectively, for VBRM/VBRC and three-level ACDF. Complications occurred in nine patients (20.9%), with the majority of them (*n* = 7) treated with a three-level ACDF. A single subsidence that did not need a revision surgery was registered in a patient treated with a VBRM, while all the reinterventions (*n* = 3) regarded patients who underwent a VBRC instrumentation: for impending mechanical failure with evolving kyphosis in two cases and, in one patient, for a CSF leak.

### 3.3. Clinical Outcome

**Figure 1:**
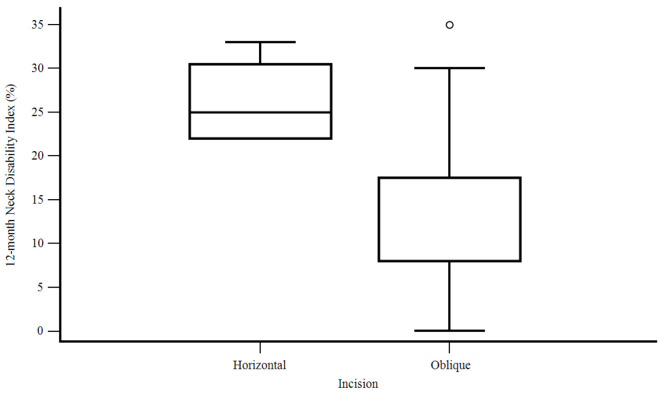
12-month Neck Disability Index in relation to the surgical approach.

Strategies using an oblique incision rather than a horizontal one positively influenced the NDI at 12 months (12.6% instead of 26.2%) (F [1, 41] = 10.296, *p* = 0.003) (Figure 1).

**Figure 2:**
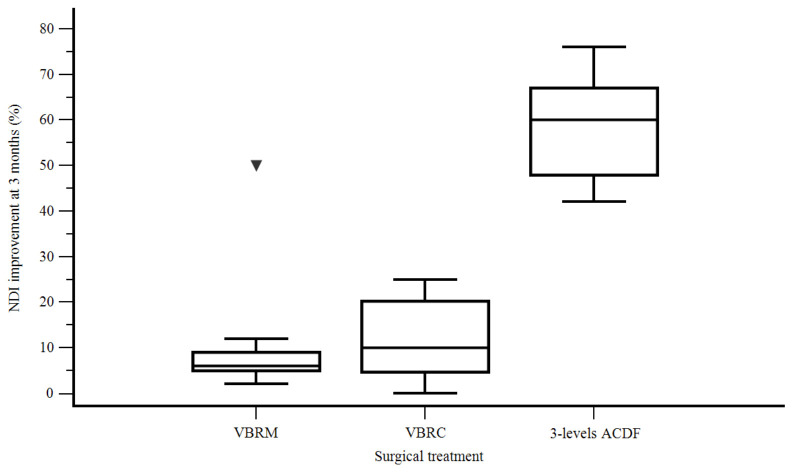
Neck Disability Index improvement at 3 months according to the surgical treatment.

**Figure 3:**
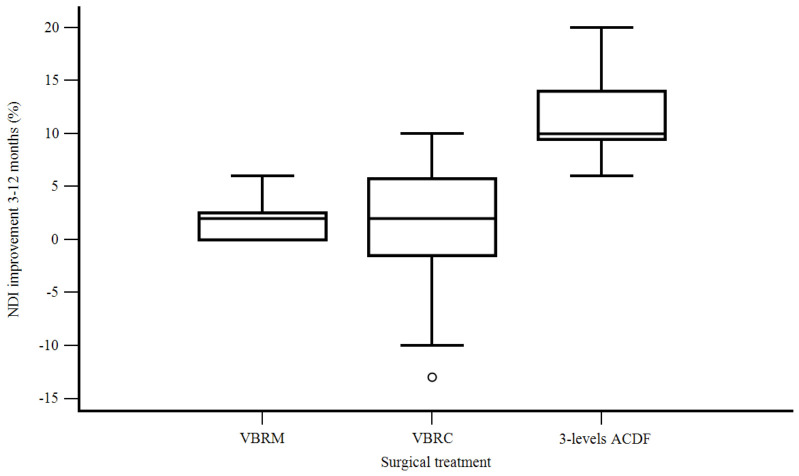
Neck Disability Index improvement between 3 and 12 months according to the surgical treatment.

Postoperatively, patients gaining >50% in terms of their NDI needed shorter hospital stays than those ones without such a neck disability recovery (3 days instead of 11.2 days) (F [1, 41] = 4.570, *p* = 0.03). A clear advantage of three-level ACDF over two-level ACCF with VBRM and VBRC was documented in terms of improvement in the NDI percentage at 3 months compared with the preoperative value (58.8% ACDF, 11% VBRM, 12% VBRC) (F [2, 40] = 92.831, *p* < 0.001) (Figure 2) and at 12 months compared with 3 months after surgery (11% ACDF, 1.1% VBRM 1.2% VBRC) (F [2, 40] = 21.707, *p* < 0.001) (Figure 3). 

**Figure 4:**
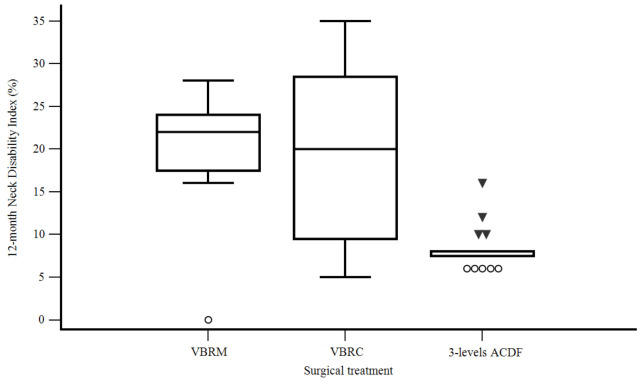
12-month Neck Disability Index according to the surgical treatment.

Overall, patients benefited more from three-level ACDF than from two-level ACCF both in terms of the NDI at 12 months (8.3% ACDF, 19.3% VBRM, 19.3% VBRC) (F [2, 40] = 12.903, *p* < 0.001) (Figure 4) and in relation to the VAS at 12 months (1.3 instead of 2.6) (F [1, 41] = 9.378, *p* = 0.004), even comparing the different techniques (Table 3). 

### 3.4. Radiological Outcome

**Figure 5:**
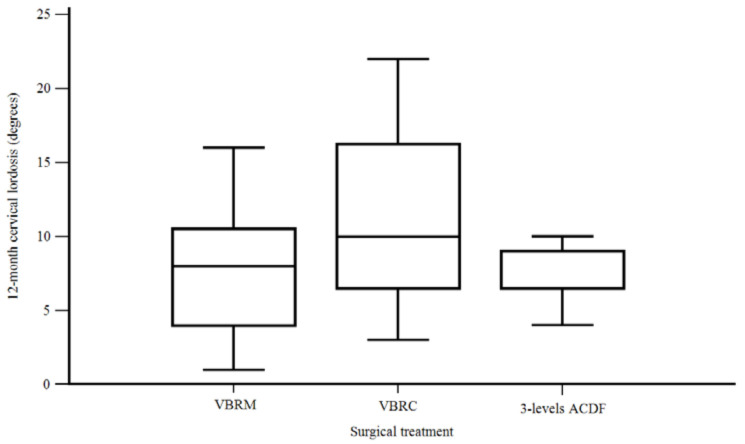
12-month cervical lordosis according to the surgical treatment.

VBRC allowed for better cervical lordosis values at 12 months (CL-fup) compared with VBRM and three-level ACDF, with mean measures, respectively, of 11.6°, 8°, and 7.6° (F [2, 40] = 3.822, *p* = 0.03) (Figure 5) (Table 4).

Similarly, in terms of 12-month losses of lordosis correction, a slight but significant disadvantage of three-level ACDF was recorded compared with two-level ACCF, with a mean loss of 0.5° instead of 0° (F [2, 40] = 3.771, *p* = 0.03). Stratifying patients into three age classes, better mean grades for CL-fup were registered for 50–59 years (11.6°) than for 60–69 years (7.2°) or 70–79 years (7.8°) (F [2, 40] = 3.211, *p* = 0.05), while no differences were noted regarding either CL-fup or loss of lordosis correction from the analysis of the different spine levels involved in the fusion. Values of CL-fup > 9° were prevalently associated with the double corpectomy strategy (Chi-squared, *p* = 0.002), while patients’ demographics, comorbidities, and spine levels treated did not influence the radiological outcomes. From our analysis of contingency tables, only three-level ACDF were associated with a relatively negative radiological outcome, with an increased risk of loss of lordosis correction of ≥ 1° in the logistic regression analysis (Table 5). 

## 4. Discussion

The treatment of three-level CSM represents a challenge to the surgeon when adequate decompression of the neural structures can only be performed using an anterior approach. Anterior surgical techniques include ACDF, ACCF, and anterior cervical hybrid decompression and fusion (HDF) (one-level corpectomy plus one-level discectomy) [16]. Our study is the first to analyze radiological and clinical outcomes distinguishing between fusion techniques adopted after double corpectomy (VBRM or VBRC) and presenting an ACDF group without plating. We believe that some of the controversial results of our study depend, in part, on the different vertebral body replacement devices adopted in ACCFs and on the avoidance of the anterior plate in ACDF procedures. More specifically, different from other authors—who found that three-level ACDF was associated with lower complication rates, less blood loss, and greater cervical lordosis compared with two-level ACCF [16,17,22,23,24]—we faced more complications with ACDF. We recorded similar blood loss rates in the three treatment strategies and better cervical lordosis angles with VBRC, with three-level ACDF representing a risk factor for lost lordosis correction at 12 months. The latter result is probably due to the choice of always mounting plates with maximum degrees of lordosis on the expandable cages in the VBRC group. In this sense, the relatively small gap between the VBRM and VBRC in terms of lordosis correction could be the effect of relative underperformance in the expandable cages we used, which are probably designed more for single corpectomies and thus are not always able to completely restore a lordosis adequate to the levels treated after a double corpectomy. Moreover, different from other authors, we had only two patients with degenerative kyphotic deformities needing an ACCF, so in most cases, the initial cervical sagittal alignment did not require extreme lordosis correction [16,23]. On the other hand, the absence of an anterior plate in our ACDF group could have played a role in terms of loss of lordosis correction, probably because this strategy is preferred for elderly patients; without the support provided by an anterior plate, slight subclinical subsidence in the cages was favored [25,26,27]. In this field—that is, in terms of completing the fixation process with an anterior plate in both ACDF and ACCF—Lau et al., did not observe significant differences in postoperative cervical lordosis or complication rates between the two groups [15]. Despite the loss of correction at follow-up, the primary stability obtained when performing multiple-level ACDFs was high enough not to need a postoperative collar [28]. Conversely, with a soft collar, cervical immobilization after either VBRM or VBRC was usually prescribed. This aspect has to be carefully considered since this external orthosis has been progressively re-evaluated in clinical practice because of its side effects [29]. Controversially, in other studies, no differences in intraoperative blood loss were noted between two-level ACCF and three-level ACDF [15,16,22]. This result is likely related to the use of three-level ACDFs on elderly and comorbid patients having an increased risk of bleeding. Although it does not provide optimal radiological outcomes, two-level ACCF with VBRM was revealed to be surprisingly safe and effective based on data comparable to the VBRC group in terms of clinical outcomes, with only one minor complication reported and with hospitalization times as long as the ACDF group. Although complications were mainly recorded after ACDF, this procedure led to better results than ACCF at 12 months in terms of the VAS and the NDI. In fact, while transient dysphagias and one wound infection after ACDF were resolved with conservative treatments, the major complications documented after ACCF required reoperations, resulting in prolonged neck pain and functional limitations [30,31]. Compared with horizontal incisions, oblique ones were associated with better NDI values at 12 months, likely thanks to the wider exposure provided, which decreased both the time of the pharyngo-esophageal retraction and the retraction force. However, with a relatively limited patient sample, we did not identify any correlation between radiological and clinical outcomes. In this sense, an advantage for three-level ACDF was recognizable, especially in more fragile patients, with an acceptable loss of lordosis correction at 12 months that did not impact their positive clinical outcomes. The latter result was more influenced by complications than by the restoration of a satisfactory lordosis: similar results in terms of 12-month VASs and NDIs were registered in the VBRM and VBRC group, with expandable cages providing better lordosis correction than mesh implants but at the cost of a higher percentage of major complications. The positive influence of an adequate correction, counterbalanced by the negative influence of major complications, showed a relative underperformance in VBRCs compared with VBRMs in terms of clinical outcome. This finding raises a new point of discussion, especially in the most fragile patients, on whether a complete correction of the sagittal deformity should be pursued even with high-risk procedures or if it is more appropriate to use safer surgeries but accept lower reliability in preserving the correction of the lordosis. As such, patient subgroup hybrid constructs are more likely to represent the right compromise, providing better segmental stability and lordosis than two-level ACCFs, but with less bleeding and a lower complication rate than the latter procedure and less loss of correction than a three-level ACDF without plating [16]. 

### Limitations

The retrospective design of this study influenced the level of evidence of the present investigation, and the relatively small patient sample has to be considered for a correct analysis of data, together with the preference for treating elderly and fragile patients with three-level ACDFs among all the surgeons. Moreover, the follow-up time could be shorter than needed for evaluating long-term outcomes, and since this was a multicenter study with procedures performed by different surgeons, the surgical techniques and the indication for a specific procedure were not standardized.

## 5. Conclusions

Our results suggest that three-level ACDF is associated with better clinical outcomes in terms of quality of life and neck pain, while two-level ACCF is associated with greater CL corrections but with a higher risk of complications. Although three-level ACDF was an independent risk factor for loss of lordosis correction, this strategy should be preferred for elderly and comorbid patients, being the least invasive of all anterior decompressions. More investigations are needed to evaluate the relationship between radiological and clinical outcomes, verifying how important it is to correct cervical lordosis to obtain benefits in terms of quality of life. Further age-related investigations are needed, focused on the role of anterior plating and different surgical strategies in providing appropriate cervical lordosis corrections in avoiding major complications and in determining advantages in terms of clinical outcomes and quality of life. 

## Figures and Tables

**Table 1:** Clinical features of the 43 patients who underwent three-level ACDF and two-level ACCF (VBRM and VBRC).

Characteristics	n = 43
Age, median (range)	68 y (53–80 y)
Male: Female (%)	n = 26:17 (60.5%:39.5%)
ASA 1	n = 6 (14.0%)
2	n = 27 (62.8%)
3	n = 10 (23.3%)
Myelopathy (yes: no)	n = 37:6 (86%:14%)
Osteoporosis (yes: no)	n = 16:27 (37.2%:62.8%)
Diabetes (yes: no)	n = 11:32 (25.6%:74.4%)
Smoking status (yes: no)	n = 16:27 (37.2%:62.8%)
Surgery: 3-levels ACDF	n = 21 (48.8%)
2-levels ACCF with VBRM	n = 9 (20.9%)
2-levels ACCF with VBRC	n = 13 (30.2%)

**Table 2:** Preoperative clinical and radiological values.

Preoperative Values	Surgical Treatment			*p*-Values
	Three-Level ACDF Mean ± SD	Two-Level ACCF with VBRM Mean ± SD	Two-Level ACCF with VBRC Mean ± SD	
VAS-pre	7.4 ± 1.1	6.3 ± 2	6.4 ± 2.7	0.22
NDI-pre	78.2% ± 10.2%	32% ± 9.7%	32.9% ± 12.3%	<0.001
CL-pre	6.3° ± 2.7°	6.7° ± 4.7°	10.4° ± 6.2°	0.03

**Table 3:** Functional outcome according to the surgical treatment.

Follow-Up	Index	Surgical Treatment			*p*-Values
		Three-Level ACDF Mean ± SD	Two-Level ACCF with VBRM Mean ± SD	Two-Level ACCF with VBRC Mean ± SD	
3 months (-post)	VAS	3.8 ± 1.3	3.5 ± 1.4	3.3 ± 1.5	0.54
	NDI	19.4% ± 3.8%	21.1% ± 9.3%	20.4% ± 10%	0.83
12 months (-fup)	VAS	1.3 ± 1.1	3.3 ± 1.7	2.1 ± 1.3	0.002
	NDI	8.3% ± 2.3%	19.3% ± 8%	19.3% ± 10.8%	<0.001

**Table 4:** Radiological outcomes based on surgical treatments.

Cervical Lordosis	Surgical Treatment			*p*-Values
	Three-Level ACDF Mean ± SD	Two-Level ACCF with VBRM Mean ± SD	Two-Level ACCF with VBRC Mean ± SD	
3 months (-post)	8.2° ± 2°	7.9° ± 4.5°	11.5° ± 6.2°	0.06
12 months (-fup)	7.6° ± 2°	8° ± 4.5°	11.6° ± 6.2°	0.03

**Table 5:** Logistic regression analysis of the factor affecting radiological outcomes.

Logistic Regression Analysis for Loss of Lordosis Correction (0° or ≥1°)	*p*	OR	95% CI for OR Lower	95% CI for OR Upper
Three-level ACDF	0.05	4.522	0.983	20.796

## Data Availability

Raw data will be provided on when requested after obtaining the approval from the IRB.
